# (*E*)-2-(3,5-Di­meth­oxy­benzyl­idene)indan-1-one

**DOI:** 10.1107/S2414314620007592

**Published:** 2020-06-12

**Authors:** Elvia Encarnacion-Thomas, Roger D. Sommer, Ajay Mallia, Joseph Sloop

**Affiliations:** a School of Science and Technology, H-3209, Georgia Gwinnett College, 1000 University Center Lane, Lawrenceville, GA 30043, USA; b North Carolina State University, Molecular Education, Technology, and Research Innovation Center, 2620 Yarbrough Dr., Raleigh, NC 27695, USA; Goethe-Universität Frankfurt, Germany

**Keywords:** crystal structure, green synthesis, indanone, chalcone

## Abstract

The title chalcone, C_18_H_16_O_3_, was prepared by a solventless base-promoted Claisen–Schmidt condensation and, upon recrystallization from ethanol, obtained in 56% yield. The dihedral angle between the indanone ring system and the benzene ring is 2.54 (4) ° and the C atoms of the methoxy groups deviate from the benzene ring by 0.087 (1) and 0.114 (1) Å. In the crystal, π-stacking is the predominant inter­molecular force, with the mol­ecules stacking into columns running parallel to the *b* axis of the unit cell.

## Abstract 2

The title compound was prepared *via* a Claisen-Schmidt condensation through a solventless, green synthesis technique. The resulting crystals formed in the monoclinic space group *P*2_1_/*c*, and adopting the common *E* configuration.

## Graphical abstract

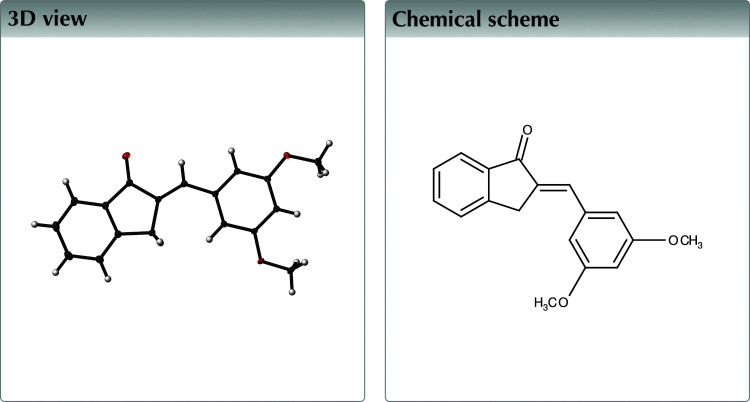

## Structure description

The chalcone family of compounds possess an aromatic α,β-unsaturated ketone functionality and can readily be formed by base-promoted condensation–dehydrations of an aromatic aldehyde and an aromatic ketone. They are important pharmacophore scaffolds and can possess anti-inflammatory, anti-fungal, anti-cancer, and anti-malarial biological activities (Singh *et al.*, 2015 ▸, 2014 ▸; Berthelette *et al.*, 1997 ▸). Additionally, the aromatic groups can be functionalized so as to produce other biological effects. The indanone family of compounds are biologically active compounds that are involved in steroid hormone biosynthesis and arachidonic acid metabolism pathways (Berthelette *et al.*, 1997 ▸). In addition, indanone derivatives serve as scaffolds for a variety of heterocycles (Sloop *et al.*, 2002 ▸, 2012 ▸).

**Figure 1:**
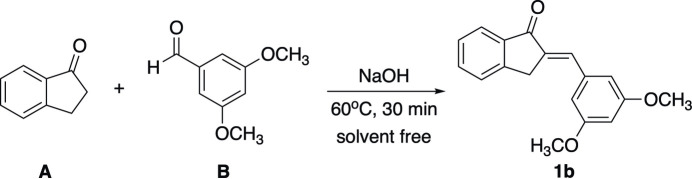
Green synthesis scheme for indanone-based chalcones

The combination of these two potential pharmacophores using greener and more efficient synthesis pathways en route to a series of highly functionalized indanone-based chalcones is now being studied by our research group. The solvent-free Claisen–Schmidt reaction undertaken in Fig. 1 ▸ minimizes reaction toxicity, limits waste production and enables easier product isolation in many cases.

**Figure 2:**
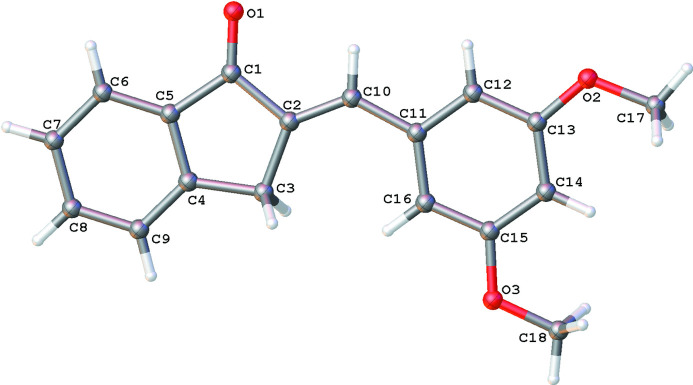
Displacement ellipsoid plot of 1b. Ellipsoids are drawn at the 50% probability level.

In the title mol­ecule (Fig. 2 ▸), the dihedral angle between the indanone ring system and the benzene ring is 2.54 (4) ° and the C‘7 and C18 atoms of the methoxy groups deviate from the benzene ring by 0.087 (1) and 0.114 (1) Å, respectively. No unusual bond lengths or angles are noted after a routine *Mogul* geometry check (Bruno *et al.*, 2004 ▸).

**Figure 3:**
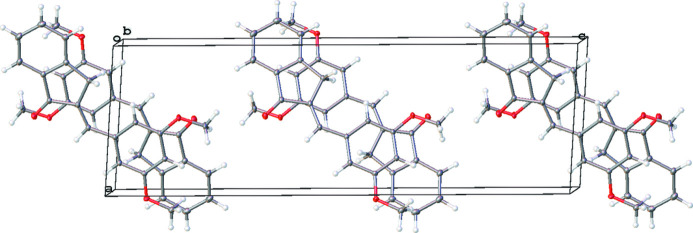
Packing diagram of 1b viewed along the *b* axis.

The predominant supra­molecular feature of this structure (Fig. 3 ▸) are slipped stacking inter­actions. This consists of ring-over-atom pairings between the indanone ring and the 3-position of the di­meth­oxy­phenyl ring of a neighboring mol­ecule and generates a relatively close contact of 2.7 Å for the methyl­ene H atoms of the indanone ring to the adjacent mol­ecule.

Structurally characterized 1b is consistent with known structures of similar indaneones. A search of the Cambridge Structural Database (Version 5.41, update of November 2019; Groom *et al.*, 2016 ▸) gave 35 hits with a similar core structure. A defined three-dimensional parameter search on the distance between the carbonyl O atom and the phenyl ring gave a clear indication of the stereochemistry of the double bond. The title compound adopts the more common *E* isomer – along with 33 of the other structures published – indicated by an O—C distances 4.2 to 4.5 Å. Only two examples of *Z* isomers (O—C of 3.2 to 3.4 Å) exist [POWZUX (Zhou *et al.*, 2009 ▸) and HAVLAR (Mori & Maeda, 1994 ▸)]. The latter has seven structure determinations as part of a light-driven solid-state isomerization study (Harada *et al.*, 2009 ▸).

## Synthesis and crystallization

A 25 mL beaker equipped with a stir bar was charged with 3,5-di­meth­oxy­benzaldehyde (0.50 g, 3.0 mmol) and warmed to 60°C. To the liquified aldehyde was added 1-indanone (0.40 g, 3.0 mmol) and solid NaOH (0.20 g, 3.8 mmol). The reaction mixture was stirred for 30 minutes at 60°C. The resulting reaction mixture was neutralized with 4 mL of 1 *M* HCl, the resulting residue was washed with several 1 mL aliquots of distilled water and the crude product (0.80 g, 95% yield) isolated *via* vacuum filtration. Recrystallization from 95% ethanol solution *via* slow evaporation afforded the target chalcone, (*E*)-2-(3,5-di­meth­oxy­benzyl­iden­yl)-1-indanone (1b) as colorless needles, (0.47 g, 56% yield). Melting range: 174–175°C. IR, ^1^H and ^13^C NMR spectroscopy and single-crystal X-ray analysis (see supporting information) confirmed the product identity.

## Refinement

Crystal data, data collection and structure refinement details are summarized in Table 1 ▸.

## Supplementary Material

Crystal structure: contains datablock(s) global, I. DOI: 10.1107/S2414314620007592/bt4094sup1.cif

Structure factors: contains datablock(s) I. DOI: 10.1107/S2414314620007592/bt4094Isup2.hkl

Supporting information file. DOI: 10.1107/S2414314620007592/bt4094Isup3.smi

Supporting information file. DOI: 10.1107/S2414314620007592/bt4094Isup4.cml

1H NMR data. DOI: 10.1107/S2414314620007592/bt4094sup5.pdf

13C NMR data. DOI: 10.1107/S2414314620007592/bt4094sup6.pdf

CCDC reference: 1894469

Additional supporting information:  crystallographic information; 3D view; checkCIF report

## Figures and Tables

**Table 1:** Experimental details

Crystal data	
Chemical formula	C_18_H_16_O_3_
*M*_r_	280.31
Crystal system, space group	Monoclinic, *P*2_1_/*c*
Temperature (K)	100
*a*, *b*, *c* (Å)	7.7611 (4), 7.2894 (4), 24.0331 (13)
β (°)	93.5573 (12)
*V* (Å^3^)	1357.02 (13)
*Z*	4
Radiation type	Mo *K*α
μ (mm^−1^)	0.09
Crystal size (mm)	0.39 × 0.12 × 0.05

Data collection	
Diffractometer	Bruker-Nonius X8 Kappa APEXII
Absorption correction	Multi-scan (*SADABS*; Krause *et al.*, 2015 ▸)
*T*_min_, *T*_max_	0.95, 0.99
No. of measured, independent and observed [*I* > 2σ(*I*)] reflections	30838, 5231, 4087
*R*_int_	0.040
(sin θ/λ)_max_ (Å^−1^)	0.772

Refinement	
*R*[*F* ^2^ > 2σ(*F* ^2^)], *wR*(*F* ^2^), *S*	0.044, 0.123, 1.02
No. of reflections	5231
No. of parameters	192
H-atom treatment	H-atom parameters constrained
Δρ_max_, Δρ_min_ (e Å^−3^)	0.61, −0.24

Computer programs: *Instrument Service*, *APEX3* and *SAINT* (Bruker, 2017 ▸), *SHELXT* (Sheldrick, 2015*a* ▸), *SHELXL2016/6* (Sheldrick, 2015*b* ▸), *Mercury* (Macrae *et al.*, 2020 ▸) and *publCIF* (Westrip, 2010 ▸).

## full crystallographic data

### Crystal data

**Table:**

C_18_H_16_O_3_	*F*(000) = 592
*M**_r_* = 280.31	*D*_x_ = 1.372 Mg m^−^^3^
Monoclinic, *P*2_1_/*c*	Mo *K*α radiation, λ = 0.71073 Å
*a* = 7.7611 (4) Å	Cell parameters from 242 reflections
*b* = 7.2894 (4) Å	θ = 3.0–33.1°
*c* = 24.0331 (13) Å	µ = 0.09 mm^−^^1^
β = 93.5573 (12)°	*T* = 100 K
*V* = 1357.02 (13) Å^3^	Needle, colourless
*Z* = 4	0.39 × 0.12 × 0.05 mm

### Data collection

**Table:**

Bruker-Nonius X8 Kappa APEXII diffractometer	5231 independent reflections
Radiation source: fine-focus sealed tube	4087 reflections with *I* > 2σ(*I*)
Graphite monochromator	*R*_int_ = 0.040
Detector resolution: 8.3333 pixels mm^-1^	θ_max_ = 33.3°, θ_min_ = 2.6°
phi and ω scans	*h* = −11→11
Absorption correction: multi-scan (SADABS; Krause *et al.*, 2015)	*k* = −11→11
*T*_min_ = 0.95, *T*_max_ = 0.99	*l* = −37→37
30838 measured reflections	

### Refinement

**Table:**

Refinement on *F*^2^	Primary atom site location: structure-invariant direct methods
Least-squares matrix: full	Secondary atom site location: difference Fourier map
*R*[*F*^2^ > 2σ(*F*^2^)] = 0.044	Hydrogen site location: inferred from neighbouring sites
*wR*(*F*^2^) = 0.123	H-atom parameters constrained
*S* = 1.02	*w* = 1/[σ^2^(*F*_o_^2^) + (0.0695*P*)^2^ + 0.2851*P*] where *P* = (*F*_o_^2^ + 2*F*_c_^2^)/3
5231 reflections	(Δ/σ)_max_ = 0.001
192 parameters	Δρ_max_ = 0.61 e Å^−^^3^
0 restraints	Δρ_min_ = −0.24 e Å^−^^3^

### Special details

**Table:**

Geometry. All esds (except the esd in the dihedral angle between two l.s. planes) are estimated using the full covariance matrix. The cell esds are taken into account individually in the estimation of esds in distances, angles and torsion angles; correlations between esds in cell parameters are only used when they are defined by crystal symmetry. An approximate (isotropic) treatment of cell esds is used for estimating esds involving l.s. planes.
Refinement. All hydrogen atoms were seen in the difference map of later refinements, but were placed at calculated positions and refined using a riding model, setting isotropic displacement parameters to 1.2 or 1.5 times that of the parent atom for ring H atoms and methyl groups respectively.

### Fractional atomic coordinates and isotropic or equivalent isotropic displacement parameters (Å^2^)

**Table:**

	*x*	*y*	*z*	*U*_iso_*/*U*_eq_	
O1	0.47855 (9)	0.17474 (11)	0.33135 (3)	0.01698 (15)	
O2	1.03362 (8)	0.10918 (10)	0.58143 (3)	0.01475 (14)	
O3	0.48827 (9)	0.32457 (10)	0.64602 (3)	0.01472 (14)	
C1	0.37431 (12)	0.23058 (12)	0.36368 (4)	0.01126 (16)	
C2	0.40164 (11)	0.24728 (12)	0.42550 (4)	0.01007 (15)	
C3	0.23699 (11)	0.31671 (12)	0.44870 (4)	0.01069 (16)	
H3A	0.191109	0.227118	0.474981	0.013*	
H3B	0.256199	0.435489	0.468075	0.013*	
C4	0.11611 (11)	0.33843 (12)	0.39744 (4)	0.01048 (16)	
C5	0.19615 (12)	0.29325 (12)	0.34906 (4)	0.01129 (16)	
C6	0.11094 (12)	0.30860 (13)	0.29642 (4)	0.01457 (18)	
H6	0.168019	0.279979	0.263676	0.017*	
C7	−0.05951 (13)	0.36685 (14)	0.29328 (4)	0.01710 (19)	
H7	−0.120329	0.378944	0.257935	0.021*	
C8	−0.14259 (12)	0.40795 (13)	0.34182 (4)	0.01618 (18)	
H8	−0.260325	0.444422	0.339062	0.019*	
C9	−0.05538 (12)	0.39621 (12)	0.39403 (4)	0.01333 (17)	
H9	−0.111664	0.427002	0.426756	0.016*	
C10	0.55633 (12)	0.20333 (12)	0.44993 (4)	0.01049 (16)	
H10	0.63798	0.160868	0.425033	0.013*	
C11	0.62038 (11)	0.20978 (12)	0.50842 (4)	0.00944 (15)	
C12	0.79305 (11)	0.15891 (12)	0.52012 (4)	0.01034 (15)	
H12	0.86179	0.122965	0.490626	0.012*	
C13	0.86425 (11)	0.16081 (12)	0.57468 (4)	0.01032 (15)	
C14	0.76564 (11)	0.21385 (12)	0.61855 (4)	0.01067 (16)	
H14	0.813948	0.214673	0.655848	0.013*	
C15	0.59412 (11)	0.26573 (12)	0.60630 (4)	0.01004 (15)	
C16	0.52038 (11)	0.26358 (12)	0.55215 (4)	0.01045 (15)	
H16	0.40318	0.298291	0.544834	0.013*	
C17	1.10933 (12)	0.09692 (13)	0.63704 (4)	0.01411 (17)	
H17A	1.229061	0.054895	0.636091	0.021*	
H17B	1.043717	0.009564	0.658384	0.021*	
H17C	1.107152	0.217881	0.654776	0.021*	
C18	0.54517 (13)	0.30032 (15)	0.70314 (4)	0.01596 (18)	
H18A	0.57173	0.170604	0.710108	0.024*	
H18B	0.453887	0.339485	0.726921	0.024*	
H18C	0.648975	0.374224	0.711613	0.024*	

### Atomic displacement parameters (Å^2^)

**Table:**

	*U*^11^	*U*^22^	*U*^33^	*U*^12^	*U*^13^	*U*^23^
O1	0.0152 (3)	0.0258 (4)	0.0101 (3)	0.0022 (3)	0.0025 (2)	−0.0029 (3)
O2	0.0093 (3)	0.0234 (3)	0.0114 (3)	0.0048 (2)	−0.0004 (2)	−0.0017 (2)
O3	0.0125 (3)	0.0249 (4)	0.0069 (3)	0.0053 (3)	0.0018 (2)	−0.0017 (2)
C1	0.0120 (4)	0.0128 (4)	0.0089 (4)	−0.0009 (3)	0.0004 (3)	−0.0003 (3)
C2	0.0117 (4)	0.0111 (4)	0.0074 (3)	−0.0006 (3)	0.0009 (3)	−0.0002 (3)
C3	0.0112 (4)	0.0120 (4)	0.0090 (3)	0.0004 (3)	0.0015 (3)	−0.0008 (3)
C4	0.0108 (4)	0.0096 (3)	0.0110 (4)	−0.0010 (3)	0.0002 (3)	0.0002 (3)
C5	0.0119 (4)	0.0119 (4)	0.0099 (4)	−0.0007 (3)	−0.0007 (3)	0.0004 (3)
C6	0.0155 (4)	0.0170 (4)	0.0109 (4)	−0.0010 (3)	−0.0017 (3)	0.0006 (3)
C7	0.0164 (4)	0.0177 (4)	0.0165 (4)	−0.0009 (3)	−0.0053 (3)	0.0025 (3)
C8	0.0121 (4)	0.0142 (4)	0.0218 (5)	0.0007 (3)	−0.0021 (3)	0.0023 (3)
C9	0.0116 (4)	0.0126 (4)	0.0159 (4)	0.0006 (3)	0.0012 (3)	0.0014 (3)
C10	0.0117 (4)	0.0116 (4)	0.0083 (3)	0.0002 (3)	0.0012 (3)	−0.0007 (3)
C11	0.0100 (4)	0.0097 (3)	0.0086 (3)	−0.0002 (3)	0.0006 (3)	−0.0004 (3)
C12	0.0110 (4)	0.0116 (4)	0.0086 (3)	0.0012 (3)	0.0016 (3)	−0.0005 (3)
C13	0.0089 (4)	0.0112 (4)	0.0109 (4)	0.0009 (3)	0.0007 (3)	−0.0002 (3)
C14	0.0103 (4)	0.0125 (4)	0.0092 (4)	0.0011 (3)	0.0004 (3)	−0.0005 (3)
C15	0.0101 (4)	0.0121 (4)	0.0080 (4)	0.0006 (3)	0.0017 (3)	−0.0010 (3)
C16	0.0094 (4)	0.0127 (4)	0.0093 (4)	0.0011 (3)	0.0003 (3)	−0.0004 (3)
C17	0.0123 (4)	0.0165 (4)	0.0132 (4)	0.0014 (3)	−0.0025 (3)	−0.0011 (3)
C18	0.0172 (4)	0.0238 (5)	0.0071 (4)	0.0020 (3)	0.0022 (3)	−0.0007 (3)

### Geometric parameters (Å, º)

**Table:**

O1—C1	1.2255 (11)	C8—H8	0.95
O2—C13	1.3673 (11)	C9—H9	0.95
O2—C17	1.4289 (11)	C10—C11	1.4623 (12)
O3—C15	1.3665 (11)	C10—H10	0.95
O3—C18	1.4267 (11)	C11—C16	1.4006 (12)
C1—C5	1.4777 (13)	C11—C12	1.4020 (12)
C1—C2	1.4929 (12)	C12—C13	1.3912 (12)
C2—C10	1.3421 (12)	C12—H12	0.95
C2—C3	1.5127 (13)	C13—C14	1.3952 (12)
C3—C4	1.5098 (13)	C14—C15	1.3975 (12)
C3—H3A	0.99	C14—H14	0.95
C3—H3B	0.99	C15—C16	1.3890 (12)
C4—C5	1.3913 (12)	C16—H16	0.95
C4—C9	1.3935 (12)	C17—H17A	0.98
C5—C6	1.3951 (12)	C17—H17B	0.98
C6—C7	1.3869 (14)	C17—H17C	0.98
C6—H6	0.95	C18—H18A	0.98
C7—C8	1.3999 (15)	C18—H18B	0.98
C7—H7	0.95	C18—H18C	0.98
C8—C9	1.3908 (13)		
			
C13—O2—C17	117.69 (7)	C2—C10—H10	114.6
C15—O3—C18	118.00 (7)	C11—C10—H10	114.6
O1—C1—C5	126.64 (8)	C16—C11—C12	119.40 (8)
O1—C1—C2	126.83 (8)	C16—C11—C10	123.99 (8)
C5—C1—C2	106.53 (7)	C12—C11—C10	116.61 (8)
C10—C2—C1	118.94 (8)	C13—C12—C11	120.34 (8)
C10—C2—C3	132.21 (8)	C13—C12—H12	119.8
C1—C2—C3	108.84 (7)	C11—C12—H12	119.8
C4—C3—C2	103.34 (7)	O2—C13—C12	115.58 (8)
C4—C3—H3A	111.1	O2—C13—C14	123.71 (8)
C2—C3—H3A	111.1	C12—C13—C14	120.70 (8)
C4—C3—H3B	111.1	C13—C14—C15	118.42 (8)
C2—C3—H3B	111.1	C13—C14—H14	120.8
H3A—C3—H3B	109.1	C15—C14—H14	120.8
C5—C4—C9	119.79 (8)	O3—C15—C16	115.30 (8)
C5—C4—C3	111.71 (8)	O3—C15—C14	122.96 (8)
C9—C4—C3	128.50 (8)	C16—C15—C14	121.73 (8)
C4—C5—C6	121.84 (8)	C15—C16—C11	119.40 (8)
C4—C5—C1	109.53 (8)	C15—C16—H16	120.3
C6—C5—C1	128.63 (8)	C11—C16—H16	120.3
C7—C6—C5	118.09 (9)	O2—C17—H17A	109.5
C7—C6—H6	121.0	O2—C17—H17B	109.5
C5—C6—H6	121.0	H17A—C17—H17B	109.5
C6—C7—C8	120.48 (9)	O2—C17—H17C	109.5
C6—C7—H7	119.8	H17A—C17—H17C	109.5
C8—C7—H7	119.8	H17B—C17—H17C	109.5
C9—C8—C7	121.00 (9)	O3—C18—H18A	109.5
C9—C8—H8	119.5	O3—C18—H18B	109.5
C7—C8—H8	119.5	H18A—C18—H18B	109.5
C8—C9—C4	118.77 (9)	O3—C18—H18C	109.5
C8—C9—H9	120.6	H18A—C18—H18C	109.5
C4—C9—H9	120.6	H18B—C18—H18C	109.5
C2—C10—C11	130.87 (8)		
			
O1—C1—C2—C10	2.20 (14)	C3—C4—C9—C8	179.35 (9)
C5—C1—C2—C10	−178.24 (8)	C1—C2—C10—C11	178.55 (9)
O1—C1—C2—C3	−178.27 (9)	C3—C2—C10—C11	−0.85 (17)
C5—C1—C2—C3	1.28 (9)	C2—C10—C11—C16	1.67 (15)
C10—C2—C3—C4	179.41 (10)	C2—C10—C11—C12	−178.08 (9)
C1—C2—C3—C4	−0.03 (9)	C16—C11—C12—C13	0.36 (13)
C2—C3—C4—C5	−1.36 (9)	C10—C11—C12—C13	−179.87 (8)
C2—C3—C4—C9	179.02 (9)	C17—O2—C13—C12	−175.86 (8)
C9—C4—C5—C6	1.74 (14)	C17—O2—C13—C14	4.54 (13)
C3—C4—C5—C6	−177.92 (8)	C11—C12—C13—O2	−179.86 (8)
C9—C4—C5—C1	−178.10 (8)	C11—C12—C13—C14	−0.25 (13)
C3—C4—C5—C1	2.24 (10)	O2—C13—C14—C15	179.29 (8)
O1—C1—C5—C4	177.40 (9)	C12—C13—C14—C15	−0.29 (13)
C2—C1—C5—C4	−2.16 (10)	C18—O3—C15—C16	169.35 (8)
O1—C1—C5—C6	−2.42 (16)	C18—O3—C15—C14	−11.69 (13)
C2—C1—C5—C6	178.02 (9)	C13—C14—C15—O3	−178.18 (8)
C4—C5—C6—C7	−1.47 (14)	C13—C14—C15—C16	0.72 (13)
C1—C5—C6—C7	178.33 (9)	O3—C15—C16—C11	178.37 (8)
C5—C6—C7—C8	−0.26 (14)	C14—C15—C16—C11	−0.61 (13)
C6—C7—C8—C9	1.73 (15)	C12—C11—C16—C15	0.06 (13)
C7—C8—C9—C4	−1.46 (14)	C10—C11—C16—C15	−179.69 (8)
C5—C4—C9—C8	−0.24 (13)		
