# Supplementary material for: (E)-2-(3,5-Di­meth­oxy­benzyl­idene)indan-1-one
Source: IUCrdata. 2020 Jun 12;5(Pt 6):x200759. doi: 10.1107/S2414314620007592 (PMC9462231; doi:10.1107/S2414314620007592)
Supplement: Supplementary file 5 [file x-05-x200759-sup5.pdf]

This report was created by ACD/NMR Processor Academic Edition. For more information go to www.acdlabs.com/nmrproc/

1/25/2019 3:14:51 PM

Acquisition Time (sec)	2.5608	***Comment***	STANDARD FLUORINE PARAMETERS			***Date***	Jan 25 2019
Date Stamp	Jan 25 2019	***File Name***	G:\MY DRIVE\SHULTZ GROUP\HEWITT\DATA\SLOOP COMPOUND\SLOOP_1HNMR_CDCL3.FID\FID				
Frequency (MHz)	399.87	***Nucleus***	1H	***Number of Transients***	64	***Original Points Count***	16384
Points Count	16384	***Pulse Sequence***	s2pul	***Receiver Gain***	46.00	***Solvent***	CHLOROFORM-d
Spectrum Offset (Hz)	2407.4006	***Spectrum Type***	STANDARD	***Sweep Width (Hz)***	6397.95	***Temperature (degree C)***	AMBIENT TEMPERATURE

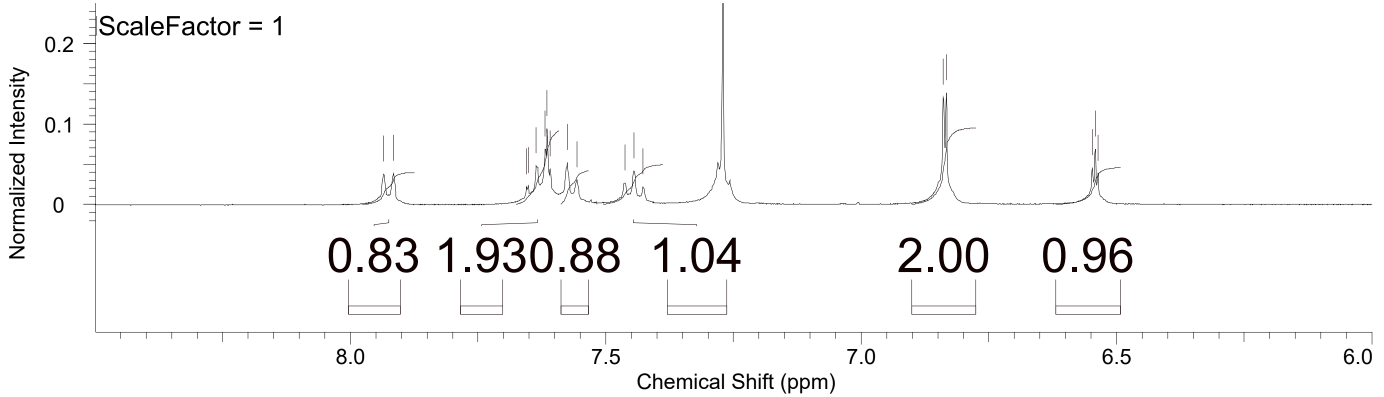

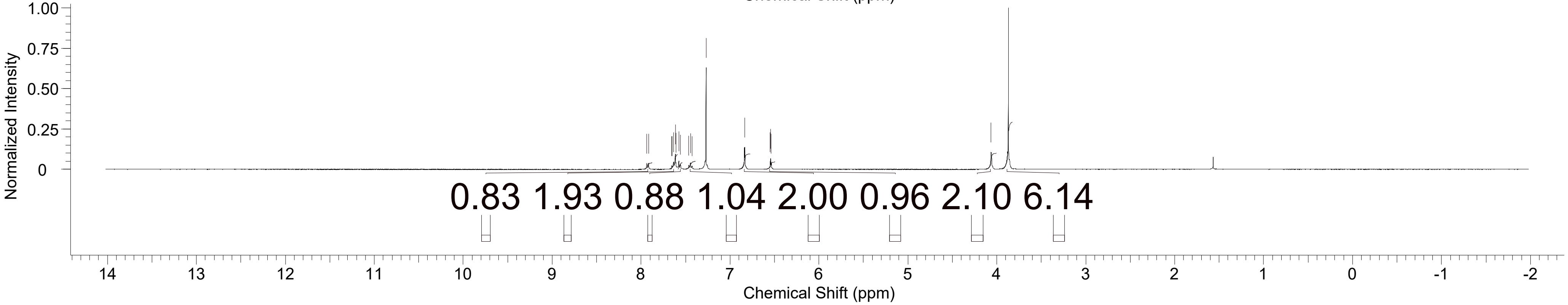

No.	(ppm)	Value	Absolute Value	Non-Negative Value
1	[3.8224 .. 3.94	6.14056396	7.04576320e+8	6.14056396
2	[4.0043 .. 4.13	2.09750366	2.40670288e+8	2.09750366
3	[6.4930 .. 6.61	0.95928574	1.10069696e+8	0.95928574
4	[6.7751 .. 6.90	1.99662209	2.29095024e+8	1.99662209
5	[7.3880 .. 7.50	1.03906095	1.19223208e+8	1.03906095
6	[7.5339 .. 7.58	0.87936789	1.00899816e+8	0.87936789
7	[7.5922 .. 7.67	1.92547262	2.20931232e+8	1.92547262
8	[7.8743 .. 7.97	0.82563263	9.47341600e+7	0.82563263

This report was created by ACD/NMR Processor Academic Edition. For more information go to www.acdlabs.com/nmrproc/

No.	(ppm)	(Hz)	Height
1	7.94	3173.0	0.0376
2	7.92	3165.2	0.0386
3	7.65	3060.9	0.0222
4	7.65	3059.8	0.0233
5	7.64	3053.5	0.0482
6	7.62	3046.5	0.0687
7	7.61	3044.9	0.0945
8	7.61	3042.6	0.0437
9	7.58	3029.3	0.0520
10	7.56	3021.5	0.0307
11	7.46	2984.0	0.0265
12	7.44	2977.0	0.0416
13	7.43	2970.0	0.0216
14	7.27	2907.1	0.6323
15	6.84	2734.9	0.1334
16	6.83	2732.5	0.1383
17	6.55	2618.1	0.0457
18	6.54	2615.7	0.0683
19	6.54	2613.8	0.0386
20	4.06	1625.4	0.1064
21	3.87	1547.3	1.0000
