# Supplementary material for: (E)-2-(3,5-Di­meth­oxy­benzyl­idene)indan-1-one
Source: IUCrdata. 2020 Jun 12;5(Pt 6):x200759. doi: 10.1107/S2414314620007592 (PMC9462231; doi:10.1107/S2414314620007592)
Supplement: Supplementary file 6 [file x-05-x200759-sup6.pdf]

This report was created by ACD/NMR Processor Academic Edition. For more information go to www.acdlabs.com/nmrproc/

1/25/2019 3:04:57 PM

Acquisition Time (sec)	1.3038	***Comment***	STANDARD FLUORINE PARAMETERS			***Date***	Jan 25 2019
Date Stamp	Jan 25 2019	***File Name***	G:\MY DRIVE\SHULTZ GROUP\HEWITT\DATA\SLOOP COMPOUND\SLOOP_13CNMR_CDCL3.FID\FID				
Frequency (MHz)	100.56	***Nucleus***	13C	***Number of Transients***	940	***Original Points Count***	32768
Points Count	32768	***Pulse Sequence***	s2pul	***Receiver Gain***	30.00	***Solvent***	CHLOROFORM-d
Spectrum Offset (Hz)	11024.7813	***Spectrum Type***	STANDARD	***Sweep Width (Hz)***	25133.52	***Temperature (degree C)***	AMBIENT TEMPERATURE

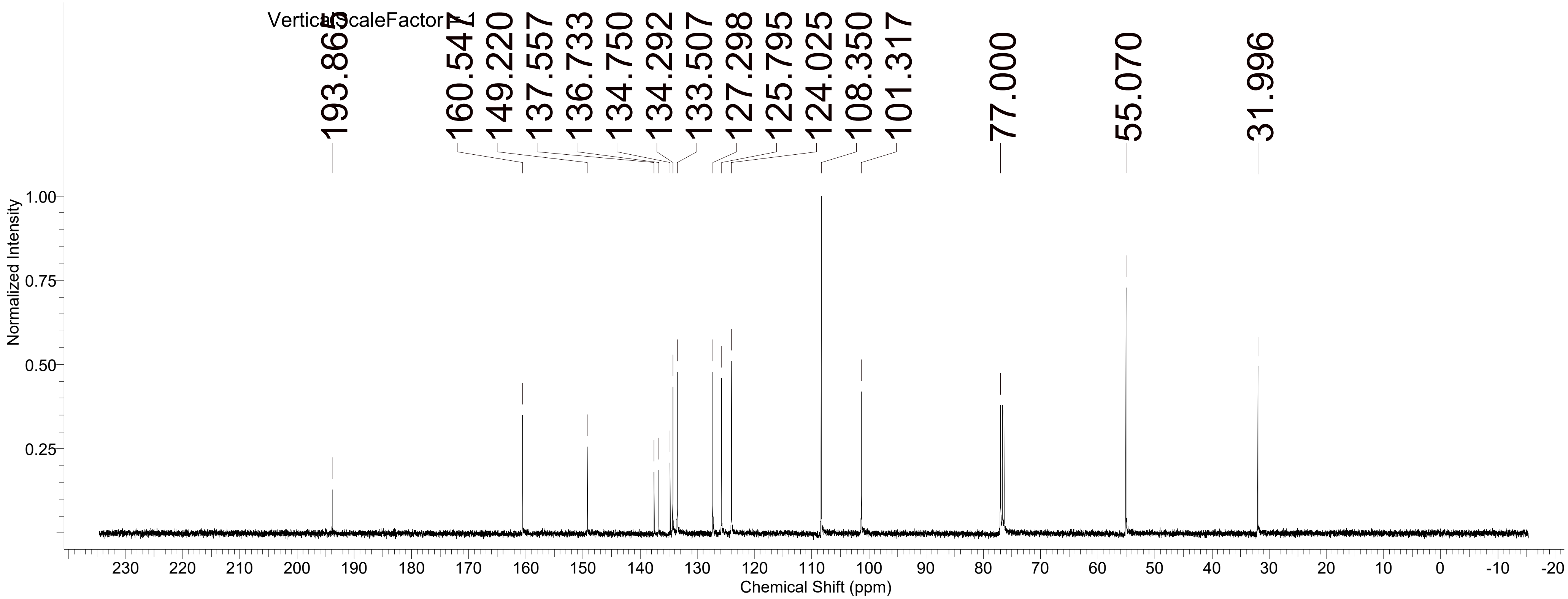

No.	(ppm)	(Hz)	Height	No.	(ppm)	(Hz)	Height	No.	(ppm)	(Hz)	Height	No.	(ppm)	(Hz)	Height
1	193.87	19494.8	0.1290	5	136.73	13749.7	0.1864	9	127.30	12800.9	0.4790	13	101.32	10188.3	0.4197
2	160.55	16144.4	0.3507	6	134.75	13550.3	0.2084	10	125.79	12649.8	0.4601	14	77.00	7743.0	0.3791
3	149.22	15005.3	0.2556	7	134.29	13504.2	0.4332	11	124.03	12471.8	0.5095	15	55.07	5537.8	0.7278
4	137.56	13832.5	0.1808	8	133.51	13425.2	0.4780	12	108.35	10895.5	1.0000	16	32.00	3217.5	0.4959
